# Metabolomic profiling and stable isotope tracing of human schwannomas: A novel perspective on tumor biology and radiation response

**DOI:** 10.1093/noajnl/vdaf223

**Published:** 2025-10-15

**Authors:** Mark C Dougherty, Hashim S Syed, Linjing Xu, S John Liu, David R Raleigh, Adam J Rauckhorst, Eric B Taylor, Marlan R Hansen

**Affiliations:** Department of Neurosurgery, Johns Hopkins University School of Medicine, Baltimore; Department of Otolaryngology—Head and Neck Surgery, University of Iowa, Iowa City; Department of Otolaryngology—Head and Neck Surgery, University of Iowa, Iowa City; Department of Radiation Oncology, University of California San Francisco, San Francisco; Department of Neurological Surgery, University of California San Francisco, San Francisco; Department of Radiation Oncology, University of California San Francisco, San Francisco; Department of Neurological Surgery, University of California San Francisco, San Francisco; Department of Cellular and Molecular Medicine, Florida International University, Miami; Department of Molecular Physiology and Biophysics, Carver College of Medicine, University of Iowa, Iowa City; Department of Otolaryngology—Head and Neck Surgery, University of Iowa, Iowa City

**Keywords:** metabolomics, NF2, radiation, schwannoma

## Abstract

**Background:**

Although schwannomas are common and benign, their growth patterns are often hard to predict. Currently, surgery and radiotherapy are the only standard treatments. Since metabolites are the end products of genes and proteins, metabolomics may reveal downstream tumor features in ways that other -omics cannot. Here, we use metabolomic profiling and stable isotope tracing to characterize primary human schwannomas and describe their changes following radiation in patient-derived xenografts.

**Methods:**

Schwannomas collected during surgical resection underwent metabolomic profiling with gas chromatography-mass spectrometry and liquid chromatography-mass spectrometry (*N* = 44) as well as DNA methylation profiling (*N* = 29). Large tumors were also implanted subcutaneously in athymic mice as patient-derived xenografts. Mice were randomized to radiation treatment or control 4-6 weeks post-implantation. Xenografts were harvested 72 h after radiation for metabolomic profiling (*N* = 53). Another group of xenografts (*N* = 33) was injected with U-^13^C-glutamine prior to tumor harvest for stable isotope tracing.

**Results:**

The schwannoma metabolome differs from that of Schwann cells, and metabolomics-based clustering of schwannomas resembles DNA methylation-based classification. In xenografts, radiation decreases cellular proliferation and produces small but detectable changes to the tricarboxylic acid (TCA) cycle and nucleotide metabolism. ^13^C-glutamine tracing shows that schwannomas can produce urea cycle intermediates, TCA cycle intermediates, cytosine monophosphate (CMP), and cytosine triphosphate from glutamine even after radiation. CMP was the only metabolite with altered ^13^C uptake following radiation.

**Conclusions:**

Schwannomas have distinct metabolic signatures compared to the Schwann cells from which they originate. Schwannoma xenograft metabolism is surprisingly robust to radiotherapy, and xenografts readily incorporate glutamine into the TCA cycle, urea cycle, and pyrimidine synthesis.

Key Points

- Metabolomic profiling clearly distinguishes schwannomas from normal Schwann cells.
- Metabolomics-based clustering closely resembles existing molecular classification.
- Schwannoma metabolism is highly robust to radiotherapy.

Importance of the Study

This is the first study to evaluate schwannoma biology using metabolomics techniques in both primary schwannomas and patient-derived xenografts, and the first to integrate metabolomic analysis with DNA methylation profiling. Here, we identify differences between Schwann cell and schwannoma metabolism, especially in nucleotide metabolism. We also find that metabolomics-based clustering significantly overlaps with molecular classification using DNA methylation profiling. Finally, using ^13^C-glutamine tracing in patient-derived xenografts, we show that schwannomas can utilize excess glutamine for metabolic needs via multiple pathways, an ability that is nearly unaffected by radiotherapy.

Schwannomas account for 8% of CNS neoplasms and are the most common tumor in the cerebellopontine angle.^1^ Although schwannomas can occur on any myelinated nerve, the most common location is on a vestibular branch of cranial nerve VIII (vestibular schwannomas or VS), and those within the cranium and spine are the most clinically important due to their propensity to cause neurological dysfunction. Uncontrolled growth can result in devastating neurological consequences, including ataxia, hemiparesis, dysphagia, and hydrocephalus in severe cases. The majority of schwannomas are sporadic, but patients with NF2-related Schwannomatosis (formerly named Neurofibromatosis type 2) develop bilateral VS as well as meningiomas and ependymomas. Surgical resection and stereotactic radiosurgery (SRS) are first-line treatments for schwannomas, but when those fail, few options remain.^2^^,^^3^ In schwannomas, SRS typically arrests growth without shrinking the tumor. Although long-term stability is a sufficient treatment goal for schwannomas, the durability (>10 years) of SRS beyond 10 years lacks data, its side effect profile is suboptimal, its use for large (>3 cm) and NF2-associated schwannomas remains highly controversial, and schwannoma radiobiology is still not fully understood.^4–6^

Although all schwannomas are benign, some are more aggressive than others, and their growth patterns remain difficult to predict. Recent studies have significantly advanced our understanding of the molecular basis for schwannoma tumorigenesis. Genetic studies suggest that most sporadic schwannomas harbor loss of NF2/Merlin function; several other mutated genes have also been identified in schwannomas, but to date, these mutations do not fully explain schwannoma tumorigenesis.^7–10^ Most recently, DNA methylation analysis and single-cell multi-omic analysis have identified 2 distinct molecular sub-groups of sporadic VS with significant clinical implications, and integration of methylome and transcriptome data suggests that these 2 molecular groups are consistent across platforms.^11–13^ Unfortunately, these advances have yet to impact clinical care.

Since metabolites are the end products of genetic processes and protein activities, metabolomics can reveal common downstream mechanisms in ways that other -omics fields often cannot. Metabolomics is the investigation of metabolism by measuring metabolite levels and pathways and rates of flux. Metabolomic profiling consists of measuring the relative concentrations of many metabolites at a given time, thus describing the steady-state, metabolomic fingerprint of a given disease state.^14^ Furthermore, stable isotope tracing using labeled metabolites—such as ^13^C-labeled glutamine or glucose—enables pathway-specific analyses to identify metabolic steps that may underlie a disease’s metabolic abnormalities.^15^ These techniques have been used to study gliomas, medulloblastomas, and meningiomas, and metabolism-targeted treatments have been shown to increase radiosensitivity in neuroblastoma and glioma.^16–20^

In addition to direct DNA damage and cell death, radiation is thought to cause metabolic oxidative stress and mitochondrial dysfunction, which can lead to a prolonged cell injury state.^21^^,^^22^ Given that SRS results in growth arrest rather than tumor death, such a prolonged injury state may characterize radiated schwannomas; indeed, such persistent oxidative stress has been described in previously radiated schwannomas by Robinett et al.^23^ but only in a small cohort of 4 tumors. Liu et al.^1^ recently described post-radiation changes to ascorbic acid, succinate, FAD, and alpha-ketoglutarate (AKG) in HEI-193 schwannoma cell lines cultures, but similar changes were only seen in AKG in primary human schwannoma cell cultures, raising concerns about the biological fidelity of the HEI-193 findings and cell cultures in general for studying schwannoma metabolism, given the importance of the *in vivo* microenvironment on cellular metabolism.^24–26^

Here, we use metabolomic profiling and stable isotope tracing to characterize primary human schwannomas and describe their metabolic changes following radiation in a patient-derived xenograft model. Our aim is 3-fold: First, to identify schwannoma-specific and molecular subtype-specific metabolic features in order to better understand the tumor phenotype; second, to describe the effect of radiation on schwannoma metabolism using patient-derived xenografts; and third, to evaluate how schwannomas utilize a key nutrient (glutamine) to resist radiotherapy.

## Methods

### Tumor Collection

**Figure 1.**
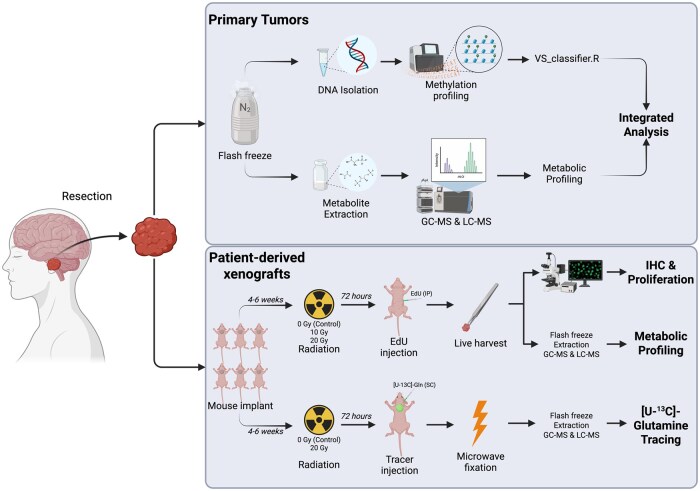
Experimental design. Created in BioRender. https://BioRender.com/1acjb7k.

All procedures involving human subjects were approved by the University of Iowa’s Institutional Review Board (IRB# 201606783). All patients presenting for surgical resection of a schwannoma by the Departments of Otolaryngology or Neurosurgery at the University of Iowa between December 2020 and April 2023 were screened for participation. Written consent was obtained from all subjects prior to tissue collection. Primary human schwannoma specimens (∼20-100 mg) were collected from acutely resected tissue and immediately flash-frozen in liquid nitrogen in the operating room. Peripheral blood samples were also collected at this time. Frozen tumor samples were stored at −80 °C until further processing. For large tumors, an additional sample was placed into ice-cold media (Hanks’ Balanced Salt Solution, Gibco #24020117, USA) and transferred to the animal facility for xenograft implantation (Figure 1). Relevant clinical data were obtained from the electronic medical record (Table S1).

### Patient-Derived Xenografts/Mouse Implantation

Acutely resected human tumors were used to establish subcutaneous xenografts in the interscapular fat pad of 8-16-week-old male athymic mice (*Foxn1^nu^/Foxn1^nu^*, Jackson Laboratory #007850). This heterotopic implantation model was previously validated and was necessary in order to obtain sufficient tissue for metabolomic and histological analysis.^27^^,^^28^ Eight to 10 mice were implanted from each primary tumor. Mice were housed under barrier conditions with unrestricted access to food until further procedures were performed. All procedures involving mice were approved by and performed in accordance with the Institutional Animal Care and Use Committee at the University of Iowa (IACUC# 1042389).

### Radiation Treatment and Harvest—Metabolomic Profiling (ie, Non-Tracer Mice)

Four to 6 weeks post-implantation, mice were randomized to receive 0 (control), 10, or 20 Gray (Gy) radiation therapy (RT). RT was delivered in a single session using the Small Animal Radiation Research Platform (Xstrahl Inc., Suwanee, GA, USA), which utilizes linear accelerator technology; single-fraction photon irradiation was chosen to mimic the most common treatment protocols used in clinical practice. Following treatment, the mice were returned to their usual housing. Starting the day before tumor harvest, 5-ethynyl-2′-deoxyuridine (EdU; A10044 Thermo Fischer Scientific) injections were administered intraperitoneally 4 times over 24 h (0, 4, 8, and 24 h) at a dose of 50 mg/kg as previously described.^29^ Tumors were harvested 72 h post-radiation under generalized inhalational anesthesia (isoflurane); a small tissue section was sharply removed and fixed in 4% paraformaldehyde for subsequent immunofluorescence and proliferation assays, and the rest was immediately flash-frozen in liquid nitrogen for metabolomic profiling. Care was taken to perform the tumor removal as quickly as possible to minimize the effects of anesthesia and tissue ischemia.^30^^,^^31^ Tumors were then stored at −80 °C until further analysis. Of note, initial pilot studies also included a second time point 4 weeks post-radiation to better characterize long-term treatment effects, but this was abandoned due to low tumor yield and high xenograft mortality.

### Radiation Treatment and Harvest—Stable Isotope (^13^C) Tracing (ie, Tracer Mice)

Four to 6 weeks post-implantation, mice were randomized to receive 0 (control) or 20 Gy radiation as described above. Universally-^13^C-labeled glutamine ([U-^13^C]-glutamine, CLM-1822-H-PK, Cambridge Isotope Laboratories Inc.) was selected as the tracer based on preliminary data. Due to the lack of prior literature, pilot studies were performed to determine the optimal injection route and dose for glutamine, as well as the tumor harvest/euthanasia method.^32^^,^^33^ Based on pilot study results, mice were fasted starting 180 min prior to harvest, then injected with 2 subcutaneous, peri-tumoral doses of [U-^13^C]-glutamine at 400 mg/kg per dose at 120 and 60 min before tumor harvest. Mice were euthanized using *in situ* focused microwave beam fixation in order to eliminate the potential effects of anesthesia and harvest-related tissue ischemia on tumor metabolism.^34^ Harvested tumors were flash frozen in liquid nitrogen and stored at −80 °C until further analysis.

### Primary Schwann Cell Cultures

Mass spectrometry-based metabolomic analysis of tissue specimens currently requires >10 mg of tissue sample; however, no such mass of Schwann cells exists in the human body in non-pathologic conditions. While segments of normal nerves, such as the Great Auricular Nerve (GAN), are often used as control tissue in the schwannoma literature, much of the GAN mass is not Schwann cells.^35^ Hence, Schwann cell cultures were selected as the best-available control. Primary human Schwann cells were purchased (10HU-188, iXCells Biotechnologies) and cultured in Schwann Cell Growth Medium (MD-0055, iXCells Biotechnologies) per the manufacturer’s instructions. Briefly, the cells were rapidly thawed in a water bath at 37 °C, washed, re-suspended in medium, transferred to a sterile T75 flask pre-coated with poly-L-lysine, and incubated at 37 °C in a humidified incubator with 5% CO_2_. One-half media volume was replaced every 48 h per the manufacturer’s recommendations. Upon reaching >90% confluence, the cells were passaged and re-plated onto 9 identical 3 cm dishes pre-coated with poly-L-lysine. Upon reaching approximately 90% confluence again, the dishes were rapidly washed with ice-cold PBS and water and flash-frozen in liquid nitrogen; approximately 45 s elapsed between media removal and freezing for each dish. The frozen dishes were stored at −80 °C until further processing.

### DNA Isolation, Methylation Profiling, and Molecular Classification (Primary VS Only)

Genomic DNA was isolated from frozen primary VS using the QIAamp Fast DNA Tissue Kit (Qiagen #51404) per the manufacturer protocol. Genome-wide methylation profiling was obtained using the Illumina MethylationEPIC 850K bead array. DNA methylation profiles were used to classify primary VS samples into neural crest (NC) or immune-enriched (IE) subtypes as described in Liu et al.^1^ (https://github.com/liujohn/schwannoma/blob/main/vs_classifier.R).

### Data Acquisition

Once all samples were obtained, frozen primary schwannoma, Schwann cell culture, and non-tracer xenograft tumor samples underwent metabolomic profiling with gas chromatography-mass spectrometry and liquid chromatography-mass spectrometry (LC-MS) as a single batch. Similarly, [U-^13^C]-glutamine tracer analysis was performed with LC-MS on xenograft tumors harvested from mice injected with glutamine tracer. Immunohistochemistry and EdU proliferation assessment were performed on fixed xenograft tissues. Full details of these procedures are provided in the online supplementary material, Methods.

### Statistics and Integrative Analysis

Data filtering, clustering, hierarchical clustering, and heatmap creation were performed using MetaboAnalyst 6.0 (metaboanalyst.ca); metabolites with >10% missing data were excluded. Statistical analysis was performed using GraphPad Prism. For primary schwannomas and Schwann cell cultures, a significance threshold of *P* < .1 with False Detection Rate adjustment was used, along with Fold Change > 2.0. The Shapiro-Wilk test was used to test for normality (Alpha = 0.05); non-normal metabolites were log transformed. Paired comparisons were deemed significant at *P* < .05 using unpaired *t*-test or Welch’s *t*-test for equal and unequal SDs, respectively.

For statistical analysis of schwannoma xenograft metabolomic profiling, Pearson correlation was used to identify the top 25 metabolites associated with radiation dose. Next, metabolite concentrations were normalized in a patient-specific fashion by dividing each value by the mean concentration of the untreated (control) xenografts that were derived from the same primary tumor. Outliers were identified and removed using Grubb’s test (Alpha = 0.01). The Shapiro-Wilk test was used to test for normality (Alpha = 0.05); non-normal metabolites were log transformed. Significance was defined as *P* < .05 using ordinary one-way ANOVA and Welch’s ANOVA for equal and unequal SDs, respectively; paired comparisons (eg, 0 vs 10 Gy) were deemed significant at *P* < .05 using uncorrected Fisher’s LSD or unpaired *t*-test with Welch’s correction for equal and unequal SDs, respectively.

## Results

### Schwannomas are Metabolically Distinct from Normal Schwann Cells

**Figure 2.**
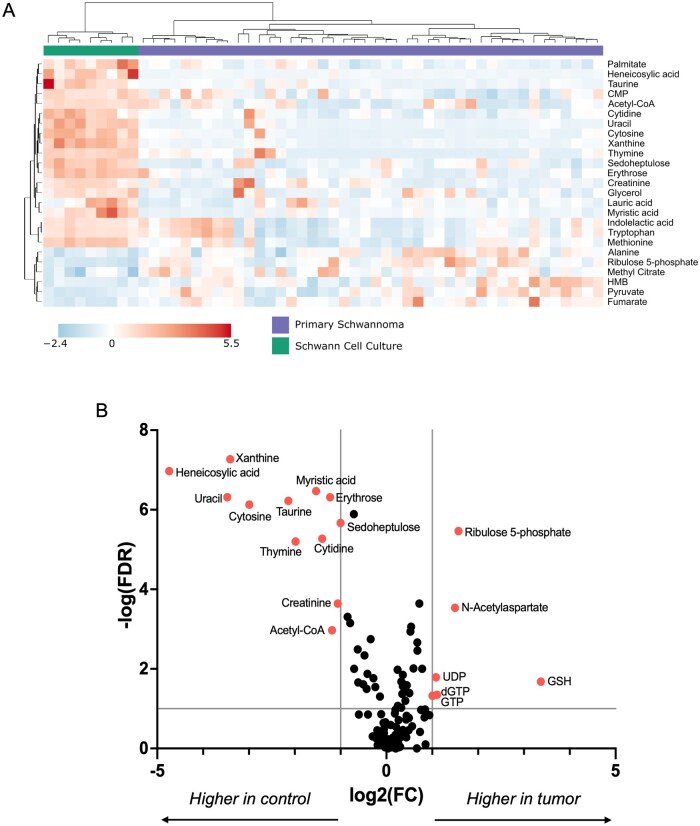
(A) Metabolomics-based unsupervised hierarchical clustering (Ward’s clustering, Euclidean distance) using the top 25 metabolites (*t*-test) clearly distinguished cultured human Schwann cells (*N* = 9) from primary schwannomas (*N* = 44). (B) Eighteen metabolites were significantly different between the Schwann cell cultures (control) and schwannomas (tumor) (threshold: Fold Change > 2.0, FDR < 0.1). FC, fold change; FDR, false detection rate.

Metabolomic profiling was performed on 44 primary human schwannomas (37 vestibular, 7 spinal) and 9 human Schwann cell cultures (Table S1). Unsupervised hierarchical clustering revealed clear metabolic differences between the 2 groups (Figure 2A). Specifically, there were 18 significant differences between the 2 groups (Figure 2B), with 12 metabolites higher in Schwann cells (xanthine, heneicosylic acid, myristic acid, erythrose, uracil, taurine, sedoheptulose, cytosine, thymine, cytidine, creatinine, and acetyl-CoA) and 6 higher in schwannomas (ribulose-5-phosphate, N-acetylaspartate, uridine diphosphate [UDP], deoxyguanosine triphosphate [dGTP], guanosine triphosphate [GTP], and reduced glutathione [GSH]).

### Metabolomic Clustering of VS Significantly Overlaps with Molecular Classification Using DNA Methylation

**Figure 3.**
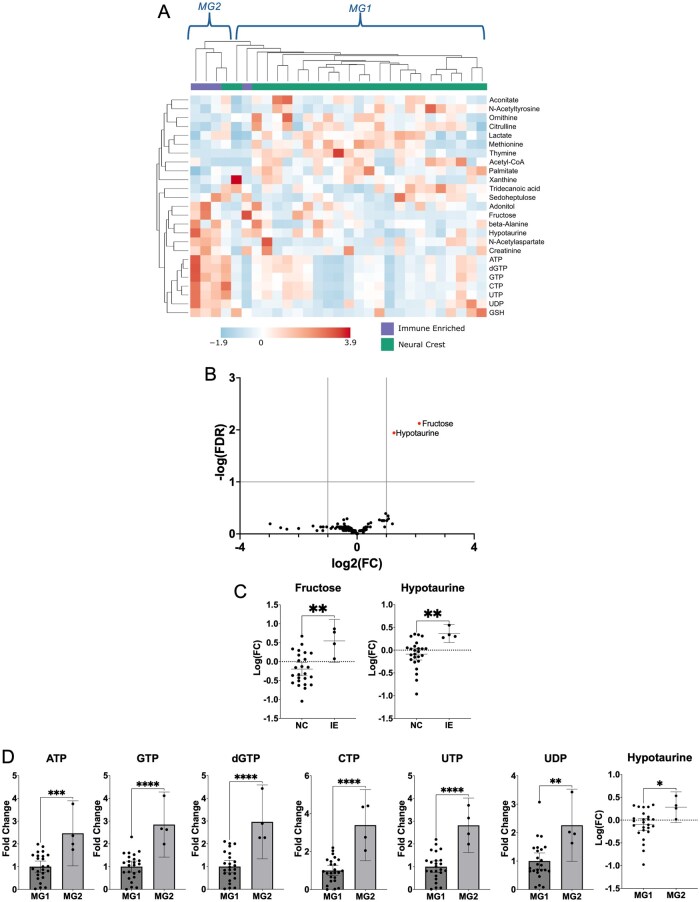
(A) Metabolomics-based unsupervised hierarchical clustering (Average clustering, Euclidean distance, top 25 features based on *t*-test) demonstrated high concordance with Liu et al.s DNA methylation-based classification of primary sporadic VS (96% of Neural Crest VS within Metabolic Group 1, 75% of Immune Enriched VS within Metabolic Group 2). (B, C) Fructose and Hypotaurine were significantly elevated in Immune Enriched VS (threshold: Fold Change > 2.0, FDR < 0.1). (D) ATP, GTP, dGTP, CTP, UTP, UDP, and Hypotaurine were significantly higher in Metabolic Group 2 (threshold: Fold Change > 2.0, FDR < 0.1). Data shown as mean ± 95% CI. *P < .05; **P < .01, ***P < .001, ****P < .0001. FC, fold change; FDR, false detection rate; IE, Immune Enriched; MG1, Metabolic Group 1; MG2, Metabolic Group 2; NC, Neural Crest.

Of the 37 primary schwannomas, DNA methylation profiling was obtained from 29 tumors. Non-VS and NF2-associated VS were omitted from DNA methylation profiling because Liu et al.s^11^ classification included sporadic VS only. Of the 29 VS profiled, there were 25 NC and 4 IE subtypes. Although the small number of the IE subtype limited statistical power, unsupervised hierarchical clustering of metabolomic data found 2 distinct metabolic groups, with 24 of the 25 NC tumors (96%) clustering with 1 IE tumor (Metabolic Group 1 [MG1]), and the other 3 IE tumors clustering with 1 NC tumor in a second group (Metabolic Group 2 [MG2]; Figure 3A). Despite the small number of tumors, fructose and hypotaurine were significantly higher in IE than NC tumors (Figure 3B and C). Taurine did not significantly differ between IE and NC tumors (*P* = .42). Notably, of the 29 profiled tumors, only 2 had previously been treated—1 with radiation and the other with surgery—both of which classified as IE and clustered with MG2. In contrast, the 1 IE tumor that clustered in MG1 had no previous treatment and no cystic changes on preoperative MRI—features more typical of NC tumors.^11^

Next, metabolites in MG1 and MG2 were compared to identify these groups’ distinguishing features (Figure 3D). Seven metabolites significantly differed: ATP, GTP, dGTP, cytosine triphosphate (CTP), UTP, UDP, and hypotaurine, all of which were higher in MG2 than MG1.

### Radiation Decreases Cellular Proliferation and Alters Several Metabolite Levels in Schwannoma Xenografts

**Figure 4.**
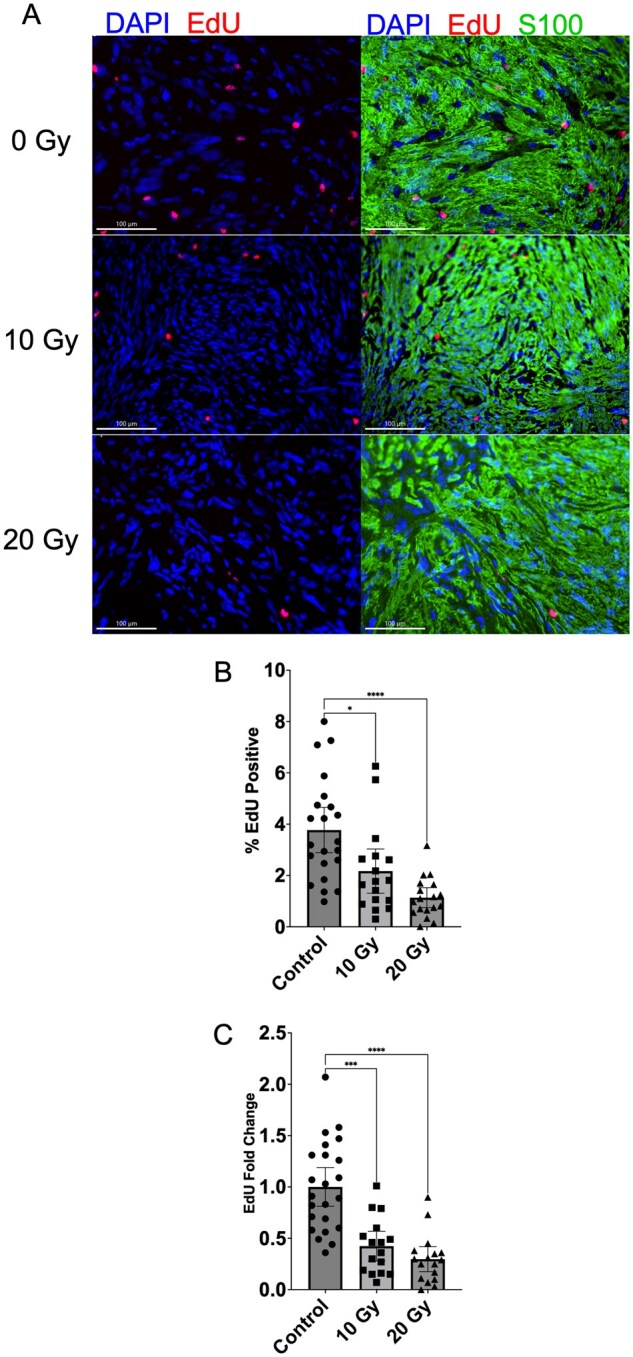
Radiation treatment decreases proliferation in schwannoma xenografts in a dose-dependent fashion. (A) EdU (red) was detected in tumor cells counterstained with DAPI and S100. (B, C) Quantification of EdU demonstrates significant reduction of EdU-positive cells with increasing radiation dose, shown as a raw percentage (B) of EdU-positive cells out of all cells, and as a fold change normalized to the control group mean of each implant group (C). Scale bar = 100 μm. Data shown as mean ± 95% CI. *P < .05; ***P < .001; ****P < .0001 (Kruskal-Wallis test).

Cellular proliferation was estimated using EdU incorporation in 61 patient-derived xenografts derived from 8 primary human schwannomas, of which there were 7 vestibular and 1 spinal schwannoma (Control: *N* = 23; 10 Gy: *N* = 19; 20 Gy: *N* = 19). There was a clear dose-dependent reduction in cellular proliferation following radiation treatment (Figure 4).

In contrast, metabolomic changes were more variable following radiation. Metabolomic profiling was obtained on 53 patient-derived xenografts derived from 7 primary human VS (Control: *N* = 19; 10 Gy: *N* = 18; 20 Gy: *N* = 16). Of these 7 VS, 1 had previously been radiated, and another was from a patient with NF2 syndrome without prior treatment on the relevant tumor; some metabolite levels appeared to be correlated with these clinical features (NF2, previous radiotherapy), but none of these correlations reached statistical significance, so further subgroup analysis was not performed (Figure S4).

**Figure 5.**
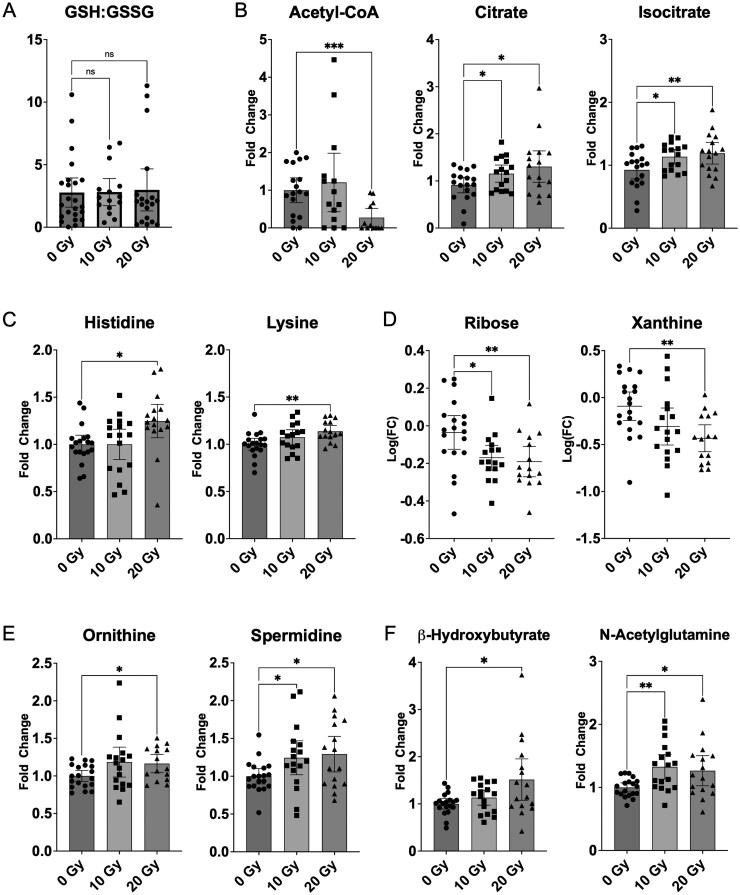
(A) Ratio of reduced to oxidized glutathione was not significantly associated with radiation dose in schwannoma xenografts. Significant metabolic alterations included (B) TCA cycle intermediates, (C) proteinogenic amino acids, (D) DNA/RNA metabolism intermediates, (E) urea cycle/polyamine pathway intermediates, and (F) beta-hydroxybutyrate and N-acetylglutamine. Data shown as mean ± 95% CI; Ribose and Xanthine data presented following logarithmic transformation. *P < .05; **P < .01; ***P < .001 (uncorrected Fisher’s LSD or unpaired *t*-test with Welch’s correction for equal and unequal SDs, respectively). GSH, reduced glutathione; GSSG, oxidized glutathione.

The ratio of reduced (GSH) to oxidized (GSSG) glutathione (GSH:GSSG) is typically decreased under conditions of oxidative stress and thus would be expected to fall after radiation treatment; however, this was not seen in the schwannoma xenografts, suggesting a relative lack of oxidative stress following radiation (Figure 5A). Among the compounds screened, there were 11 metabolites that were significantly altered by radiotherapy: acetyl-CoA, citrate, isocitrate, histidine, lysine, ribose, xanthine, ornithine, spermidine, beta-hydroxybutyrate, and N-acetylglutamine (Figure 5B-F). These suggest there are radiation-induced perturbations to the tricarboxylic acid (TCA) cycle (acetyl-CoA, isocitrate, citrate), protein biosynthesis and/or utilization (histidine, lysine), and DNA/RNA synthesis or breakdown (ribose, xanthine).

### Glutamine Carbons Contribute to TCA Cycle, Pyrimidine Synthesis, and Urea Cycle in Schwannoma Xenografts

Considering the findings from xenografts, we opted for tracing of glutamine carbons rather than glucose or lactate/pyruvate, as there were changes in amino acids (histidine, lysine) and glutamine-related pathways (TCA cycle, N-acetylglutamate) but not glycolysis (Figure 5C, E, and F). Furthermore, given the high variability of metabolites even within treatment groups, the 10 Gy treatment group was omitted, and the harvest technique was modified to reduce potential harvest-related metabolic changes. Thirty-three xenograft tumors derived from 4 primary human VS—1 of which was from an NF2 patient—were successfully harvested and analyzed following U-^13^C-glutamine injection (Control *N* = 16, 20 Gy *N* = 17).

**Figure 6.**
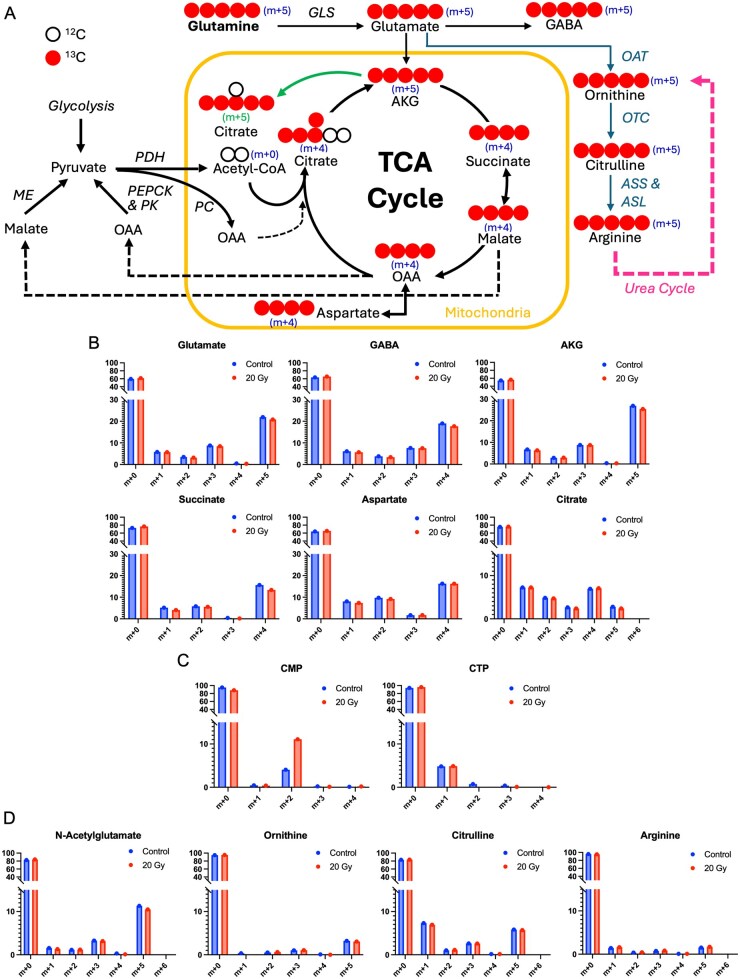
(A) Schematic of TCA cycle, urea cycle/arginine synthesis, and related metabolites; arrow from AKG to m+5 citrate indicates reductive carboxylation of AKG to Citrate. (B) TCA cycle isotopologues show significant glutamine-derived ^13^C enrichment (glutamine anaplerosis) and incorporation into glutamate and GABA. (C) Significant ^13^C enrichment is present in CMP and to a lesser extent CTP. (D) Urea cycle-related metabolites also demonstrate ^13^C enrichment, especially N-acetylglutamate and Citrulline. AKG, alpha-ketoglutarate; ASL, argininosuccinate lyase; ASS, arginosuccinate synthetase; CMP, cytosine monophosphate; CTP, cytosine triphosphate; GABA, gamma-aminobutyric acid; GLS, glutaminase; ME, malate dehydrogenase; OAT, ornithine acetyltransferase; OTC, ornithine transcarbamylase; PC, pyruvate carboxylase; PDH, pyruvate dehydrogenase; PEPCK, phosphoenolpyruvate carboxykinase; PK, pyruvate kinase; TCA, tricarboxylic acid.

^13^C enrichment was measured in 25 metabolites. More than 80% enrichment of glutamine was observed, confirming successful tracer injection (Figure S3A). At least 10% ^13^C enrichment was observed in 11 of the other 24 metabolites: glutamate, AKG, succinate, fumarate, malate, citrate, aspartate, citrulline, GABA, N-acetylglutamate, and cytosine monophosphate (CMP) (Figure 6).

^13^C enrichment in glutamate and all measured TCA cycle intermediates indicates that schwannomas can utilize excess glutamine for energetic and biosynthetic needs via TCA cycle incorporation—otherwise known as glutamine anaplerosis (Figure 6A and B). The 5 carbons from glutamine enter the TCA cycle via conversion to glutamate then to AKG, accounting for the m + 5 isotopomer of AKG. As each full turn of the TCA cycle replaces 2 carbons in AKG, the m + 3 and m + 1 isotopomers result from 1 and 2 cycle turns, respectively (Figure 6A). Similarly, since 1 labeled carbon is removed in the conversion of AKG to succinate, the m + 4 and m + 2 isotopomers of succinate, acetate/oxaloacetate, and citrate are likely to result from the first and second turns of the classical TCA cycle, respectively. In addition to the classic TCA cycle reactions, citrate, and isocitrate can also be produced from AKG via reductive carboxylation (Figure 6A), resulting in m + 5 citrate. M + 5 citrate was observed in our data (Figure 6B), suggesting that schwannoma xenografts can also make use of glutamine-derived AKG via reductive carboxylation. Surprisingly, however, ^13^C enrichment in all measured TCA cycle metabolites was similar between radiated and non-radiated xenografts, suggesting that 20 Gy radiation does not significantly impact TCA cycle activity or AKG reductive carboxylation in schwannomas.

CMP was the only measured metabolite with significant ^13^C uptake differences between radiated and non-radiated tumors (Figure 6C). Cytosine triphosphate and CMP can be produced from glutamine via *de novo* pyrimidine synthesis; however, UMP and UDP—both of which are intermediates in this pathway—do not show significant enrichment (Figure S3C), so it is not yet clear how glutamine carbons are incorporated into CTP and CMP.

Metabolites associated with the urea cycle also demonstrate enrichment, especially N-acetylglutamate and citrulline (Figure 6D). The canonical urea cycle occurs mainly in the liver, so such uptake in schwannoma cells is unexpected. This may suggest that schwannomas or other cells in the schwannoma microenvironment possess inducible extrahepatic urea cycle enzyme expression; however, the current data do not provide direct evidence of such activity.

## Discussion

This is among the first studies to investigate human schwannoma metabolism using metabolomics and—to our knowledge—the first to integrate metabolomics with DNA methylation-based molecular classification. We also describe the first stable isotope tracing protocol for patient-derived schwannoma xenografts, which adds significant technical novelty to the schwannoma literature.

### Primary Schwannomas

Clear differences were observed between schwannomas and normal Schwann cells, most notably involving nucleic acid metabolism (cytosine, cytidine, thymine, dGTP, GTP, UDP, uracil, xanthine, and ribulose-5-phosphate), fatty acids, (heneicosylic acid and myristic acid), and the pentose phosphate pathway (erythrose and sedoheptulose). These changes reflect a metabolic “fingerprint” for schwannomas, which may suggest novel biological vulnerabilities. Hence, further investigation of these pathways may reveal potential therapeutic targets.

Given recent advances in molecular characterization of schwannomas, we sought to correlate metabolomics findings with the molecular groups of Liu et al.^11^ Metabolomic and DNA methylation profiling was obtained on 29 VS; despite the limited sample size, unsupervised hierarchical clustering using metabolomics data categorized 96% of NC tumors in 1 group (MG1) and 75% (3 of 4) IE tumors in a second group (MG2), providing further evidence that Liu et al.s IE and NC groups are biologically distinct. This is particularly noteworthy given that recent meningioma literature has not identified similar concordance.^36^ We were also able to identify clinical and imaging features in our cohort that corresponded with Liu et al.s findings: Both previously treated tumors in our cohort were classified as IE and clustered with MG2, whereas the 1 IE tumor that clustered in MG1 had no prior treatment and lacked cystic changes on preoperative MRI—both features associated with NC tumors.

Despite limited samples, fructose and hypotaurine were significantly different between the IE and NC tumors. This may reflect immune-cell infiltration, as some literature suggests there is a link between fructose level and T-cell activity, as well as elevated hypotaurine in leukocytes.^37^^,^^38^ There is mounting evidence that immune cell infiltration correlates with schwannoma growth and clinical features; future work should clarify whether these distinct metabolomic signatures correlate with immune cell infiltration.^24^^,^^25^

MG2 tumors had elevated ATP, GTP, dGTP, CTP, UTP, UDP, and hypotaurine compared to MG1 tumors (Figure 3D). As most of these metabolites are high-energy nucleotide triphosphates, this would suggest that high energy availability is a defining feature of MG2. These differences may arise from differential immune cell infiltration: Liu et al.^1^ showed that IE tumors have more proliferative macrophage and lymphocyte infiltration than NC tumors, and our Metabolic Groups highly correlate with the IE/NC classification.

### Patient-Derived Xenografts

Radiotherapy significantly altered 11 metabolites in schwannoma xenografts (Figure 5); these may implicate TCA cycle activity (acetyl-CoA, isocitrate, citrate), protein metabolism (histidine, lysine), and DNA/RNA metabolism (ribose, xanthine) in the response of schwannomas to radiation. What’s more, ornithine and spermidine, both of which increased post-radiation, may indicate flux through the polyamine pathway via ornithine transcarbamylase, the products of which are necessary for DNA double-strand break repair.^39^ Increased beta-hydroxybutyrate is associated with fasting or mitochondrial dysfunction.^40^ Finally, N-acetylglutamine may be protective in the setting of ischemia or protein deficiency.^41^

Liu et al.described metabolomic profiling in schwannoma cell cultures, identifying increased ascorbate and decreased succinate, FAD, and AKG following radiation in HEI-193 cell line cultures and decreased AKG in primary human schwannoma cell cultures. In the current study, none of these 4 metabolites reached significance following radiation, but ascorbate (*P* = .0667) and succinate (*P* = .2857) trended in the same direction as Liu et al.s^11^ observations, while AKG trended in the opposite direction (*P* = .1859).

Despite the prevalence of SRS as a treatment modality, schwannomas are relatively radioresistant; growth rate typically slows after SRS, but tumors rarely decrease in size, and viable cells persist.^4^^,^^23^ Our findings are consistent with these features: First, schwannomas grow very slowly, reflected in the low proliferation rate in unirradiated tumors (4%); second, radiation significantly reduced the proliferation rate but did not lead to apparent tumor demise (Figure 4). Furthermore, persistent c-Jun N-terminal kinase activity has previously been linked to schwannoma resistance to oxidative stress-mediated damage even after radiation.^28^^,^^42–44^ In the current study, radiation did not change the GSH:GSSG ratio in schwannoma xenografts, suggesting a lack of radiation-induced oxidative stress, further in keeping with previously published findings in VS (Figure 5A).

### [U-^13^C]-Glutamine Tracing

Significant ^13^C enrichment of glutamate, GABA, and TCA cycle intermediates indicates that schwannomas readily route glutamine carbons into the TCA cycle via glutamate and AKG (glutamine anaplerosis). In addition to the classical TCA cycle, schwannomas can produce citrate and isocitrate via reductive carboxylation of AKG, as shown by the presence of m + 5 citrate (Figure 6B). Given the centrality of the TCA cycle in cellular metabolism, such activity suggests that schwannoma cells can widely utilize environmental glutamine for both energy and biosynthetic substrate.

^13^C enrichment was also observed in metabolites associated with the urea cycle and arginine biosynthesis, especially N-acetylglutamate and citrulline (Figure 6D). N-acetylglutamate is a necessary cofactor for the first step of the urea cycle, while ornithine, citrulline, and arginine are all urea cycle intermediates that can be synthesized from glutamine via glutamate. The canonical urea cycle occurs primarily in the liver and serves to dispose of toxic excess ammonia (NH_3_) produced by amino acid catabolism. What’s more, several cancers demonstrate a metabolic hallmark termed Urea Cycle Dysregulation, wherein urea cycle enzymatic activity is altered to increase pyrimidine production, resulting in elevated pyrimidine:purine ratios.^45^ Hence, ^13^C enrichment of these 4 metabolites (Figure 6D) may indicate that schwannomas can induce extrahepatic urea cycle activity to counteract ammonia buildup, increase pyrimidine synthesis, or both. To further characterize these findings, future experiments could include ^13^C tracing of argininosuccinate, arginine, and ornithine, ^15^N tracing from ammonia, and evaluating urea cycle enzyme activity.

Interestingly, ^13^C enrichment was similar between 0 and 20 Gy treatment groups for most metabolites at the measured time point of 72 h. However, 20 Gy radiation did induce a shift in ^13^C uptake in CMP (Figure 6C). CMP and other pyrimidines can be produced from glutamine by the *de novo* pyrimidine synthesis pathway. Increased glutamine production is associated with radioresistance in several cancers, purportedly due to facilitating DNA damage repair via glutamine-derived pyrimidine synthesis; thus, the increased ^13^C enrichment in CMP following radiation may indicate that schwannomas use available glutamine in a similar fashion to combat radiation.^46^ However, neither UDP nor UMP—which are intermediates for glutamine-derived CMP via the *de novo* pathway—had significant ^13^C enrichment (Figure S3C), so it is not yet clear how glutamine carbons were incorporated into CTP and CMP, nor is it clear why CTP did not change with radiation in the same manner as CMP. Other measured nucleotides—AMP, GMP, UMP, and UDP—did not have significant glutamine-derived ^13^C enrichment, so their response to radiation cannot be assessed from the current data. Further investigation of nucleotide metabolism changes following radiation may be warranted.

### Future Directions

Several follow-up experiments could help clarify our findings and further explore potential therapeutic targets. First, performing ^13^C glutamine tracing experiments in radiated and non-radiated primary Schwann cell cultures could clarify whether the glutamine utilization observed here is tumor-specific or reflects underlying Schwann cell biology. Second, transcriptional analysis of glutamine metabolic regulators (such as glutamine synthetase, GLS1, GLS2, and glutamine transporter SLC1A5) using RNA-seq could help identify molecular drivers of glutamine utilization in the setting of radiation, which may reveal potential therapeutic targets. Finally, testing the effect of glutaminolysis inhibition in our model, such as with the small-molecule GLS1 inhibitor CB-839, could help determine whether glutamine metabolism is a viable target for improving schwannoma radiosensitivity.

### Limitations

Because the primary specimens are collected from surgical subjects, there is unavoidable selection bias for larger symptomatic tumors and inevitable variation in the pre-freeze ischemia time, which can introduce metabolomic inaccuracies.^30^ However, large tumors are the most clinically important, so selecting for them could be considered a strength, and care was taken to freeze tumor specimens as quickly as possible. Moreover, most tumors were collected from 2 surgeons with similar patient populations and surgical techniques. In addition, all tumors in this study were intradural (cranial or spinal); additional investigation will be needed to characterize peripheral schwannomas’ metabolism.

Another limitation is our use of primary human Schwann cell cultures as the control tissue from which we derive schwannoma-specific metabolomic features. Cell cultures cannot perfectly reproduce the *in vivo* environment of Schwann cells since they lack other cell types, such as endothelial cells, which have been shown to influence Schwann cell function via glycolysis; while this likely implicates lactate as a key feature of the Schwann cell microenvironment, it is difficult to know how this may impact the Schwann cell metabolomic profile overall.^26^ What’s more, cell culture media contain several of the measured metabolites, which could impact profiling results. However, due to technical limitations, we believe this is the best control tissue available at present, and media-related inaccuracies are likely minimal because the cultures are thoroughly washed before freezing per validated protocols.

Metabolomic-DNA methylation comparisons were limited by small sample size; in particular, of the 29 VS profiled, only 4 VS were classified as IE. In Liu et al.^11^ the 2 molecular groups were roughly equal in number, but this discrepancy is likely due to the higher proportion of recurrent tumors in that study given the strong correlation between previous treatment and IE classification. Hence, we suspect that our study would have more IE tumors if more recurrent samples were collected. Unfortunately, due to strong batch effects inherent in GC/MS- and LC/MS-derived metabolomics, all specimens must be profiled together in 1 batch, making it very difficult to obtain very large specimen counts for metabolomics and impossible to add more specimens *post-hoc*.

Since schwannoma growth and treatment response occur over a time course of years, 72 h post-treatment is very early in the relevant time course; as such, there may be later metabolomic changes in xenograft tumors that are not observed in the current study. Furthermore, the use of immunocompromised mice could underestimate the number of metabolic changes that occur because of immune cell infiltration in human schwannomas, particularly after RT, which is believed to play an important role in schwannoma biology.^13^^,^^24^^,^^25^ In addition, the heterotopic (non-orthotopic) implant location—while necessary for technical reasons—may fail to account for native tumor/nerve interactions and is also likely more hypoxic than the native tumor environment due to lack of direct blood flow to the xenograft tumors, which can also alter the global metabolic behavior of tumors.^26^^,^^47^ Finally, metabolic and radiobiological differences may exist between NF2-related and sporadic schwannomas, as well as between radiated and radiation-naïve schwannomas, but were not identified in the current study due to the small sample size (Figure S4); future investigation would benefit from greater numbers of NF2-associated and previously radiated tumors.

### Conclusions

We find that schwannomas have a distinct metabolomic fingerprint from their normal tissue counterpart, and that metabolomics-based clustering bears high resemblance to previously published molecular classifications—further validating both classification schemata. Moreover, in patient-derived xenografts, we show that radiation produces small but detectable changes within key metabolic pathways, including the TCA cycle, nucleotide metabolism, and some amino acids. Finally, we show that schwannoma xenografts utilize excess glutamine to produce urea cycle intermediates, pyrimidine nucleotides, and TCA cycle intermediates even after 20 Gy radiation, which may help explain their radioresistant nature.

## Supplementary Material

vdaf223_Supplementary_Data

## Data Availability

Data will be made available upon reasonable request.
