# Supplementary material for: Metabolomic profiling and stable isotope tracing of human schwannomas: A novel perspective on tumor biology and radiation response
Source: Neurooncol Adv. 2025 Oct 15;8(1):vdaf223. doi: 10.1093/noajnl/vdaf223 (PMC12863081; doi:10.1093/noajnl/vdaf223)
Supplement: vdaf223_Supplementary_Data [file vdaf223_supplementary_data.zip › 2025.9.17_NOA_SuppTable1.docx]

| Patient | Sex (M/F) | Age (yr) | Location | Prior Treatment | NF2 | Diabetes | Methylation Group |
| --- | --- | --- | --- | --- | --- | --- | --- |
| 1 | F | 36 | spine | No | No | No | n/a |
| 2 | M | 58 | vestibular | No | No | No | n/a |
| 3 | M | 57 | vestibular | No | No | No | Neural Crest |
| 4 | M | 48 | vestibular | No | No | No | Neural Crest |
| 5 | F | 51 | vestibular | No | No | No | Neural Crest |
| 6 | M | 60 | vestibular | No | No | No | Neural Crest |
| 7 | F | 64 | vestibular | No | No | No | Neural Crest |
| 8 | M | 63 | vestibular | No | No | No | n/a |
| 9 | F | 63 | spine | No | No | No | n/a |
| 10 | F | 47 | vestibular | No | No | No | Neural Crest |
| 11 | F | 51 | vestibular | No | No | No | Neural Crest |
| 12 | F | 58 | vestibular | No | No | No | n/a |
| 13 | F | 66 | vestibular | No | No | No | Neural Crest |
| 14 | F | 61 | spine | No | No | No | n/a |
| 15 | F | 37 | spine | No | No | No | n/a |
| 16 | F | 76 | vestibular | No | No | No | Neural Crest |
| 17 | F | 52 | vestibular | No | No | No | n/a |
| 18 | M | 64 | vestibular | No | No | No | Neural Crest |
| 19 | M | 64 | vestibular | **Radiation** | No | No | Immune Enriched |
| 20 | M | 23 | vestibular | No | No | No | Neural Crest |
| 21 | M | 66 | vestibular | No | No | **Yes** | Neural Crest |
| 22 | M | 44 | vestibular | No | No | No | n/a |
| 23 | M | 37 | vestibular | No | No | No | Neural Crest |
| 24 | M | 39 | vestibular | No | No | No | n/a |
| 25 | M | 28 | vestibular | No | **Yes** | No | n/a |
| 26 | F | 52 | vestibular | **Surgery** | No | No | Immune Enriched |
| 27 | F | 31 | vestibular | No | No | **Yes** | Neural Crest |
| 28 | M | 35 | spine | No | No | No | n/a |
| 29 | F | 61 | vestibular | No | No | No | Neural Crest |
| 30 | F | 40 | spine | No | No | No | n/a |
| 31 | F | 76 | vestibular | No | No | No | Neural Crest |
| 32 | F | 28 | vestibular | **Radiation** | **Yes** | No | n/a |
| 33 | M | 56 | vestibular | No | No | No | Neural Crest |
| 34 | M | 64 | vestibular | No | No | No | Neural Crest |
| 35 | M | 63 | vestibular | No | No | No | Neural Crest |
| 36 | M | 36 | vestibular | No | No | No | Neural Crest |
| 37 | F | 52 | vestibular | No | No | No | Immune Enriched |
| 38 | F | 72 | spine | **Surgery** | No | No | n/a |
| 39 | F | 48 | vestibular | No | No | No | Immune Enriched |
| 40 | F | 68 | vestibular | No | No | No | Neural Crest |
| 41 | M | 66 | vestibular | No | No | **Yes** | Neural Crest |
| 42 | F | 62 | vestibular | No | No | No | Neural Crest |
| 43 | M | 50 | vestibular | No | No | No | Neural Crest |
| 44 | M | 67 | vestibular | No | No | No | Neural Crest |
