# Supplementary material for: Metabolomic profiling and stable isotope tracing of human schwannomas: A novel perspective on tumor biology and radiation response
Source: Neurooncol Adv. 2025 Oct 15;8(1):vdaf223. doi: 10.1093/noajnl/vdaf223 (PMC12863081; doi:10.1093/noajnl/vdaf223)
Supplement: vdaf223_Supplementary_Data [file vdaf223_supplementary_data.zip › 2025.9.17_NOA_SwnmaMetabolomics_SuppFigLegends_Clean.docx]

## Supplemental Figure Legends

**Supplemental Figure 1.** To assess how well patient-derived xenografts (non-tracer) replicated the metabolomic profile of their corresponding primary tumors (N=7), metabolite values of control xenografts (N=19, 2-3 xenografts per primary) were normalized to the matched primary tumor value (i.e. converted to Fold Change relative to primary tumor). Fold change (FC) values were Log2-transformed and then the mean of the absolute value of the log-transformed fold change was calculated in order to equally consider changes in either direction (i.e. increase or decrease). Dotted lines correspond to 2-fold (lower line) and 10-fold (upper line) differences between xenograft controls and matched primary tumors. Out of 96 GCMS metabolites, 62 (65%) differed less than twofold and 95 (99%) differed less than tenfold (i.e. one order of magnitude) on average. Across all GCMS metabolites, the average fold change was 1.5; stated differently, GCMS-based metabolomic profiling of xenografts phenocopied the profile of the original tumor with about 1.5-fold accuracy on average. Out of 49 LCMS metabolites, 4 (8%) differed less than twofold and 31 (63%) differed less than tenfold (i.e. one order of magnitude) on average. Across all LCMS metabolites, the average fold change was 4.36.

With 65% of GCMS metabolites in xenografts falling within 2x of the matched primary tumor value, and nearly all falling within one order of magnitude (10x), GCMS-based metabolomic profiling of patient-derived schwannoma xenografts provides a reasonably accurate phenotypic model of the tumors from which they are derived. Though less accurate than GCMS, LCMS-based profiling still produced a majority (63%) of metabolite values within one order of magnitude of the index tumor, and thus also remains a reasonable tool. The higher variation in LCMS metabolites is likely due to the higher volatility of metabolites measured with LCMS as well as a much greater range of detection in LCMS machines. Due to this volatility, we suspect that LCMS metabolomics would disproportionately benefit from optimized tumor harvest methods including Focused Microwave Beam (FMB) euthanasia; notably the xenografts tested here were not harvested with FMB.

**Supplemental Figure 2.** Oxidative stress was assessed in a representative subset of non-tracer schwannoma xenografts by staining for anti-nitrotyrosine (3NT; Sigma-Aldrich #06-284). Radiation treatment was correlated with increased oxidative stress on average, but this response was highly variable between tumors. Nuclei were co-stained with DAPI and cover slips were mounted. Images were obtained with a Leica STELLARIS 5 confocal microscope (Leica Microsystems) and analyzed with Imaris 9.9.0 software (Oxford Instruments).

**Supplemental Figure 3.** Isotopologues of selected metabolites following [U-^13^C]-glutamine injection. (A) More than 80% enrichment of glutamine was observed, confirming successful tracer injection. (B) Acetyl-CoA m+2 enrichment suggests that glutamine-derived 13C is also present in pyruvate (Figure 6A), most likely derived from malate or oxaloacetate produced by glutamine anaplerosis (Figure 6A). (C) UMP and UDP—both of which are intermediates in the *de novo* pyrimidine synthesis pathway—do not show significant enrichment, so it is not yet clear how glutamine carbons are incorporated into CTP and CMP. Abbreviations: UMP, uridine monophosphate; UDP, uridine diphosphate.

**Supplemental Figure 4.** Metabolomic profiling was obtained on 53 patient-derived xenografts derived from 7 primary human vestibular schwannomas (Control: N=19; 10 Gy: N=18; 20 Gy: N=16); one primary tumor was previously radiated, another was NF2-related, and the remaining 5 were sporadic VS without previous treatment. (A) previous radiation and NF2 status were correlated with several metabolic changes on univariate analysis, but these associations were not statistically significant following covariate adjustment. (B) Heatmap of patient-derived xenografts further demonstrates relative lack of consistent findings in NF2 and previously radiated tumors, at least within the limited number of samples tested in the current study.

**Table 1.** Patient demographics, tumor location, key clinical features, and methylation classification.
