# Supplementary material for: Metabolomic profiling and stable isotope tracing of human schwannomas: A novel perspective on tumor biology and radiation response
Source: Neurooncol Adv. 2025 Oct 15;8(1):vdaf223. doi: 10.1093/noajnl/vdaf223 (PMC12863081; doi:10.1093/noajnl/vdaf223)
Supplement: vdaf223_Supplementary_Data [file vdaf223_supplementary_data.zip › 2025.9.17_NOA_SwnmaMetabolomics_SuppMethods_Clean.docx]

# Supplemental Methods

*Metabolite Extraction*

Frozen schwannoma tissue was partitioned and weighed without thawing and subsequently lyophilized overnight. Tissue samples were bead mill homogenized in 18:1 (weight/volume) extraction solvent (2:2:1 acetonitrile:methanol:water) containing a mixture of ^13^C- or ^2^H-labeled internal standards. For cell culture samples, 1.2ml of extract solvent was added to each sample and dishes were scraped for 20 seconds to detach cells; the resulting solution was transferred to 1.5ml microcentrifuge tubes. All subsequent steps were the same for frozen tissue and cell culture samples.

Samples were then rotated for 1 hour at -20°C, then centrifuged for 10 minutes at 21,000 x g, and 90 µl (GC-MS) or 200 µl (LC-MS) of the cleared metabolite extracts were transferred to autosampler vials and dried using a SpeedVac vacuum concentrator (Thermo). Dried metabolite extracts were then analyzed with mass spectrometry as described below.

*Gas Chromatography-Mass Spectrometry (GC-MS)*

For GC-MS, sample derivatization was performed as follows: First, dried metabolite extracts were reconstituted in 18 μl of 11.4 mg/ml methoxyamine (MOX) in anhydrous pyridine, vortexed for 5 minutes, and heated for 1 hour at 60°C; next, 12 μl of N,O-Bis(trimethylsilyl)trifluoroacetamide (TMS) was added to each sample, samples were vortexed for 1 minute, then heated for 30 minutes at 60°C. 1 μl of derivatized sample was injected into a Trace 1300 GC (Thermo) fitted with a TraceGold TG-5SilMS column (Thermo) operating under the following conditions: split ratio = 5:1, split flow = 6 ml/minute, purge flow = 5 ml/minute, carrier mode = Constant Flow, and carrier flow rate = 1.2 ml/minute. The GC oven temperature gradient was as follows: 80°C for 3 minutes, increasing at a rate of 20°C/minute to 280°C, and holding at a temperature at 280°C for 8 minutes. Ion detection was performed by an ISQ 7000 mass spectrometer (Thermo) operated from 3.90 to 21.00 minutes in EI mode (-70eV) using select ion monitoring (SIM). A pooled quality control (QC) sample was run at the beginning and end of the GC-MS run and at regular intervals throughout.

*Liquid Chromatography-Mass Spectrometry (LC-MS)*

For liquid chromatography-mass spectrometry (LC-MS), dried extracts were reconstituted in 20 µL acetonitrile/water (1:1 v/v) vortexed well, rotated on a rotator in -20C overnight, centrifuged, and the supernatant was transferred to LC-MS autosampler vials for analysis. LC-MS data were acquired on a Thermo Q Exactive hybrid quadrupole Orbitrap mass spectrometer with a Vanquish Flex UHPLC system. 2 µL of each prepared sample was separated using a Millipore SeQuant ZIC-pHILIC (2.1 X 150 mm, 5 µm particle size, Millipore Sigma #150460) with a ZIC-pHILIC guard column (20 x 2.1 mm, Millipore Sigma #150437). The mobile phase was comprised of Buffer A [20 mM (NH_4_)_2_CO_3_, 0.1% NH_4_OH (v/v)] and Buffer B [acetonitrile]. The chromatographic gradient was run at a flow rate of 0.150 mL/min as follows: starting at 80% B and decreasing to 20% B over 20 minutes; returning to 80% B in 0.5 minutes; and held there for 7 minutes.^1^ For high-energy and redox studies, data were obtained in the full-scan, polarity-switching mode from 1 to 20 minutes, MS data acquisition was performed in a range of m/z 70–1,000 with the resolution set at 70,000, the AGC target set at 1 × 10^6^, and the maximum injection time set at 200 ms.^1^ For ^13^C tracing studies, data were obtained in tSIM mode guided by an inclusion list of targeted metabolites with the resolution set at 70,000, the AGC target set at 5 x 10^4^, the maximum injection time set at 200 ms, the spray voltage set to 3.0 kV, the heated capillary held at 275 °C, and the HESI probe held at 350 °C. The sheath gas flow was set to 40 units, the auxiliary gas flow was set to 15 units, and the sweep gas flow was set to 1 unit. A pooled QC sample was run at the beginning and end of the LC-MS run and at regular intervals throughout.

*GC-MS and LC-MS Data Analysis*

Raw GC-MS and LC-MS data were analyzed using TraceFinder 5.1 software (Thermo Scientific). Metabolites were identified based on the University of Iowa Metabolomics Core Facility’s standard-confirmed, in-house library. NOREVA was used for signal drift correction.^2^ Data were normalized to the sum of all the measured metabolite ions in that sample. ^12^C-natural abundance correction was performed for all ^13^C-glutamine tracing experiments as previously described.^3^

*Immunohistochemistry & Cellular Proliferation in Xenografts*

Non-tracer xenograft-bearing mice were injected with EdU 50mg/kg IP four times over 24 hours prior to tissue harvest, and a portion of these tumors was collected for immunofluorescence at the time of harvest. These samples were fixed in 4% paraformaldehyde, washed in 1x phosphate-buffered saline (PBS), cryoprotected in 30% sucrose, embedded in optimum cutting temperature compound (OCT; Thermo Fisher Scientific), cryosectioned and slide-mounted at 10 µm thickness. Frozen sections were permeabilized with 0.5% Triton X-100 in PBS for 20 minutes then blocked with blocking buffer (5% goat serum, 2% bovine serum albumin, and 0.8% Triton X-100) for 30 minutes at room temperature. EdU was detected using the Click-iT EdU Kit (Alexa Fluor 647, Invitrogen #C10340) per manufacturer protocol. Next, the samples were incubated in polyclonal rabbit anti-S100 antibody (1:200; Sigma-Aldrich #S2644) dissolved in blocking buffer at 4°C overnight. Following three washes in PBS, a secondary antibody (goat anti–rabbit Alexa Fluor 546, 1:500; Invitrogen) was applied for 2 hours at room temperature. Slides were then counter-stained with DAPI (Thermo Fisher #62248), mounted with Aqua-Mount mounting medium (Thermo #13800) and cover-slipped. Images for DAPI/S100/EdU-stained slides were obtained using MetaMorph software version 7.7.1.0 (Molecular Devices, LLC) on a Leica DMIRE 2 microscope (Leica Microsystems) equipped with epifluorescence filters.

Nuclei were counted using the built-in cell counter function in MetaMorph. Cell proliferation was estimated by dividing the total number of EdU-positive/S100-positive nuclei by the number of DAPI-positive/S100-positive nuclei. A total of 4 randomly selected 20x-magnification microscopic fields per section and 2 sections per slide were counted. The final proliferation was calculated as the mean of the proliferation of all 8 fields imaged for each tumor. During cell counting, the investigator was blinded to treatment conditions.

**References**

1. Cantor JR, Abu-Remaileh M, Kanarek N, et al. Physiologic Medium Rewires Cellular Metabolism and Reveals Uric Acid as an Endogenous Inhibitor of UMP Synthase. *Cell*. 2017;169(2):258-272.e17. doi:10.1016/j.cell.2017.03.023

2. Li B, Tang J, Yang Q, et al. NOREVA: normalization and evaluation of MS-based metabolomics data. *Nucleic Acids Res*. 2017;45(W1):W162-W170. doi:10.1093/nar/gkx449

3. Yuan J, Bennett BD, Rabinowitz JD. Kinetic flux profiling for quantitation of cellular metabolic fluxes. *Nat Protoc*. 2008;3(8):1328-1340. doi:10.1038/nprot.2008.131
