# Supplementary figures and images for: Metabolomic profiling and stable isotope tracing of human schwannomas: A novel perspective on tumor biology and radiation response
Source: Neurooncol Adv. 2025 Oct 15;8(1):vdaf223. doi: 10.1093/noajnl/vdaf223 (PMC12863081; doi:10.1093/noajnl/vdaf223)

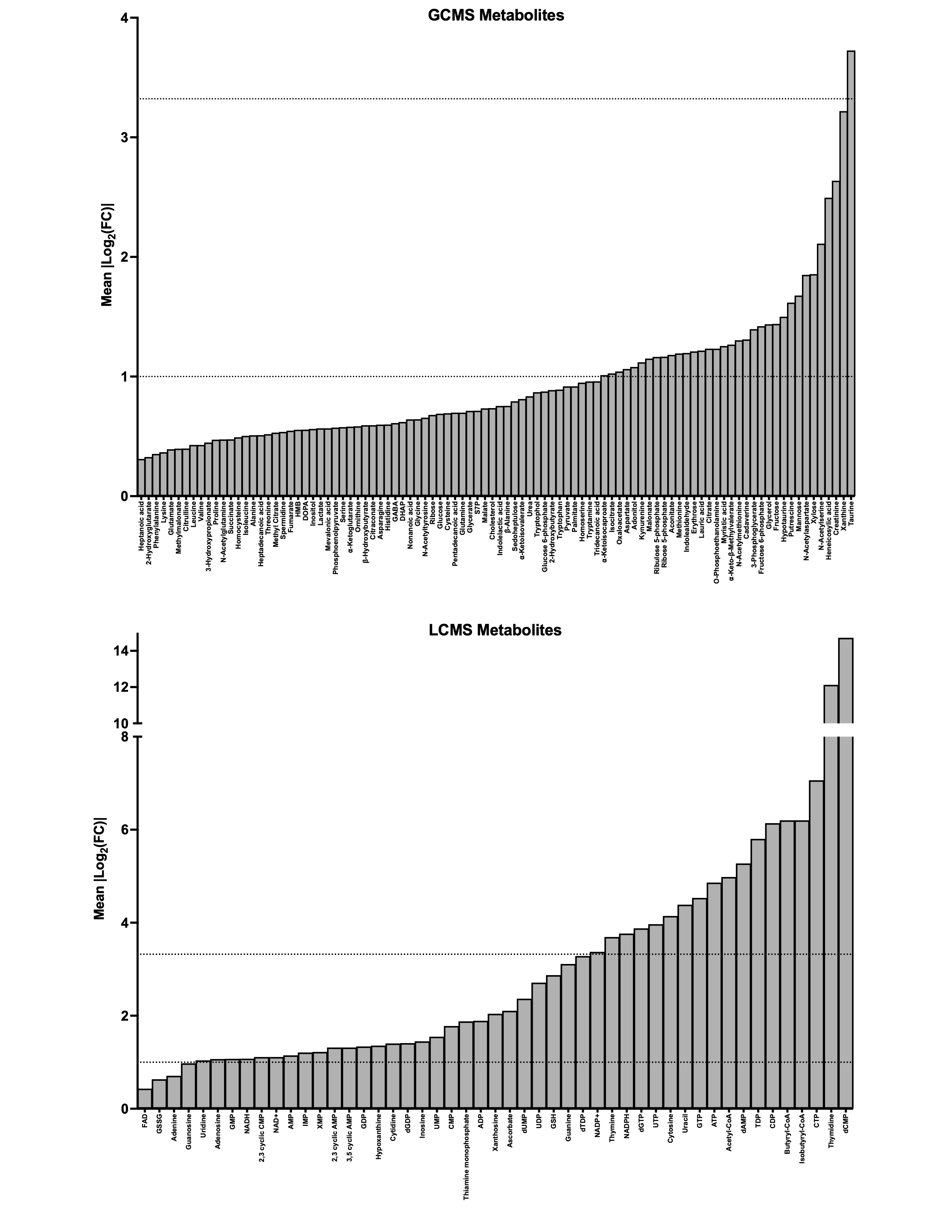

Supplement: vdaf223_Supplementary_Data [file vdaf223_supplementary_data.zip › SuppFig1_XenoCtrl vs PrimTum Phenocopy_7.15.25.tiff]

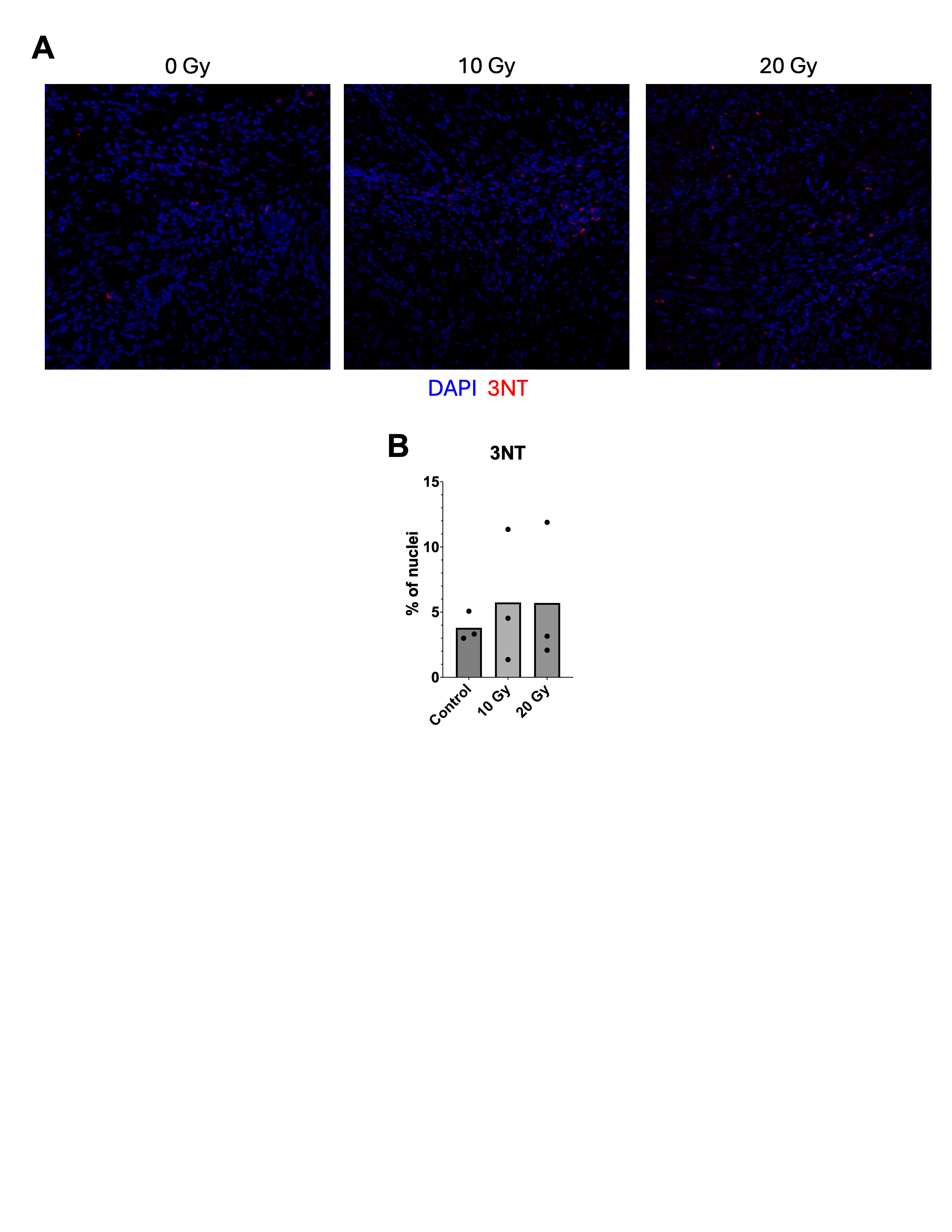

Supplement: vdaf223_Supplementary_Data [file vdaf223_supplementary_data.zip › SuppFig2_3NT_7.15.25.tiff]

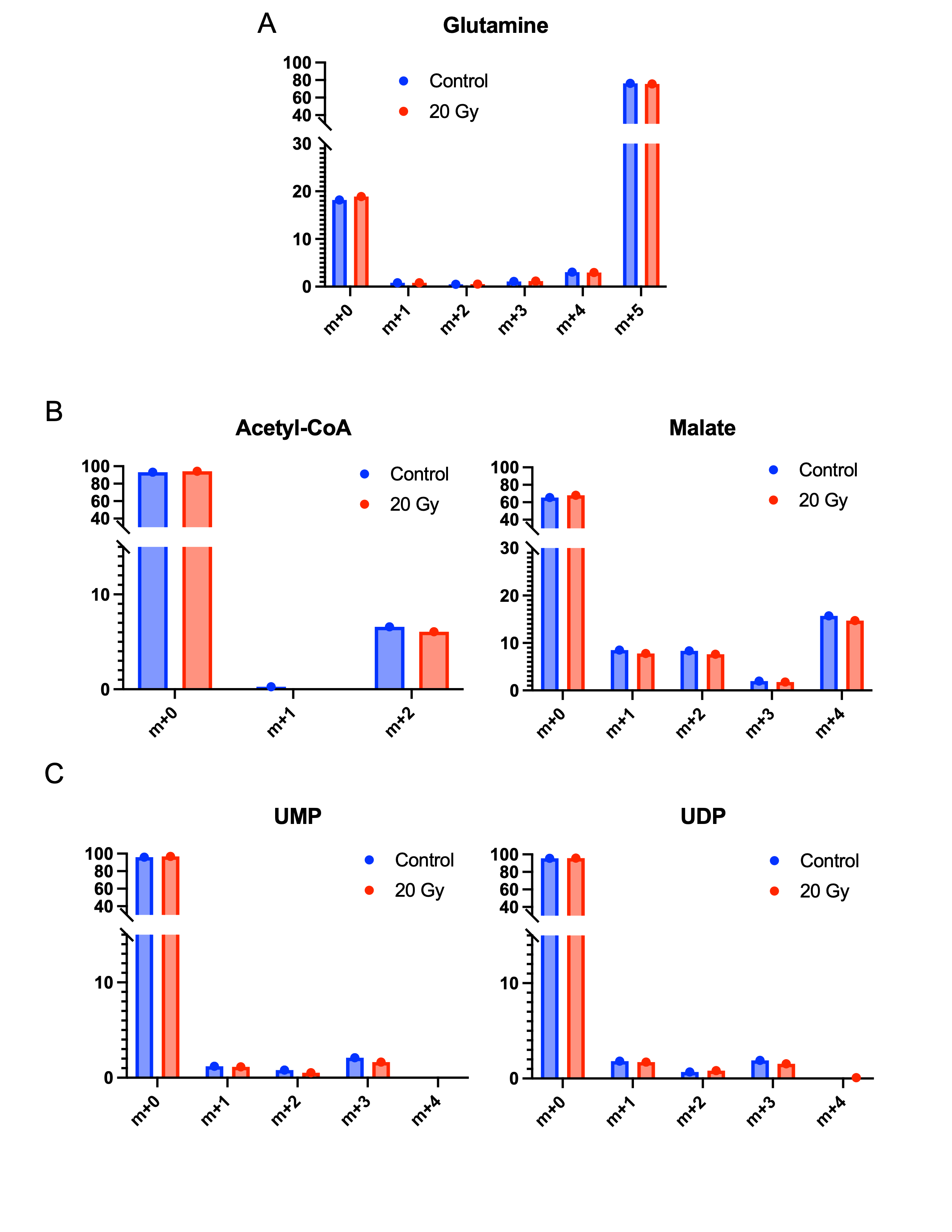

Supplement: vdaf223_Supplementary_Data [file vdaf223_supplementary_data.zip › SuppFig3_GlnTracingOther_7.15.25.tiff]

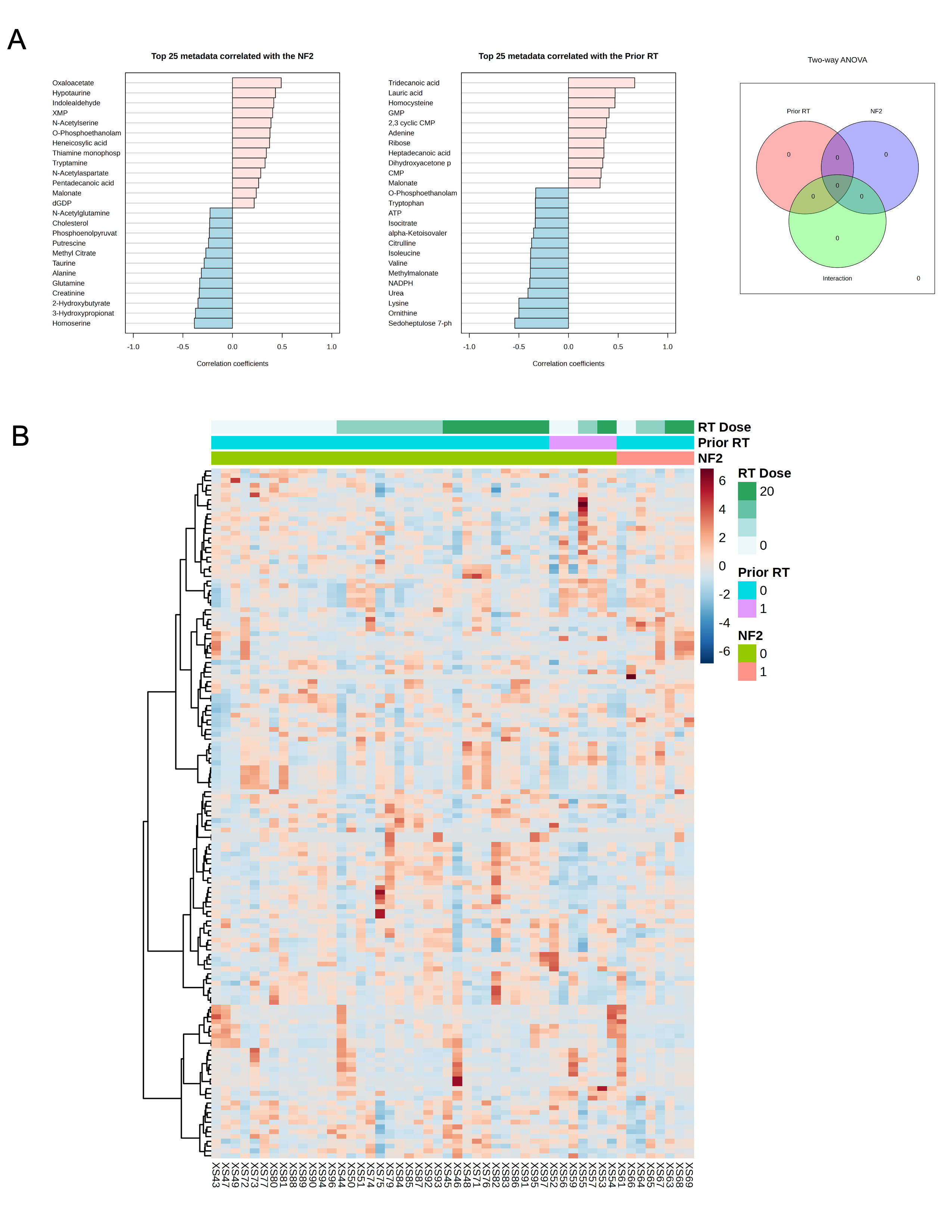

Supplement: vdaf223_Supplementary_Data [file vdaf223_supplementary_data.zip › SuppFig4_XenoNF2priorRTeffects_9.11.25.tiff]
